# Association between serum 25-hydroxyvitamin D levels and carotid atherosclerosis in chronic kidney disease patients

**DOI:** 10.1186/s12882-016-0367-7

**Published:** 2016-10-18

**Authors:** Yong-Muh Ng, Soo-Kun Lim, Pei-San Kang, Khairul Azmi Abdul Kadir, Mei-Ling Sharon Tai

**Affiliations:** 1 Division of Nephrology, Department of Medicine, Faculty of Medicine, University of Malaya, 50603 Kuala Lumpur, Malaysia; 2 Department of Family Medicine, Faculty of Medicine, University of Malaya, 50603 Kuala Lumpur, Malaysia; 3 Department of Biomedical Imaging, Faculty of Medicine, University of Malaya, 50603 Kuala Lumpur, Malaysia; 4 Division of Neurology, Department of Medicine, Faculty of Medicine, University of Malaya, 50603 Kuala Lumpur, Malaysia

**Keywords:** Vitamin D, Parathyroid hormone, Carotid atherosclerosis, Carotid intima-media thickness, Carotid plaque, Chronic kidney disease

## Abstract

**Background:**

Epidemiological studies have shown an inverse relationship between vitamin D levels and cardiovascular diseases. However, this does not infer a causal relationship between the two. Chronic kidney disease (CKD) patients have a high prevalence of vitamin D deficiency and carotid atherosclerosis. Therefore, in this study we have aimed to determine the association between serum 25-hydroxyvitamin D levels and carotid atherosclerosis in the CKD population.

**Methods:**

100 CKD stage 3–4 patients were included in the study. Direct chemiluminesent immunoassay was used to determine the level of serum 25-hydroxyvitamin D. All subjects underwent a carotid ultrasound to measure common carotid artery intima-media thickness (CCA-IMT) and to assess the presence of carotid plaques or significant stenosis (≥50 %). Vitamin D deficiency was defined as serum 25-hydroxyvitamin D < 25 nmol/L. Abnormal CCA-IMT was defined as CCA-IMT ≥ 0.8 mm. Plaque was defined as a focal structure that encroaches into the arterial lumen of ≥ 0.5 mm or 50 % of the surrounding IMT value. Significant stenosis was defined as peak-systolic velocities ≥ 125 cm/s and end-diastolic velocities ≥ 40 cm/s.

**Results:**

The vitamin D deficiency and non-deficiency groups did not differ significantly in terms of abnormal CCA-IMT (*P* = 0.443), carotid plaque (*P* = 0.349), and carotid stenosis (*P* = 0.554). No significant correlation between serum 25-hydroxyvitamin D levels and CCA-IMT (*P* = 0.693) was found. On a backward multiple linear regression model, serum 25-hydroxyvitamin D levels was not associated with CCA-IMT, abnormal CCA-IMT, or plaque presence.

**Conclusions:**

No important association between serum 25-hydroxyvitamin levels and carotid atherosclerosis was found in CKD patients.

## Background

Atherosclerosis is characterized by the accumulation of lipids and fibrous cap in the arteries [1]. It commonly affects coronary and carotid arteries. Carotid atherosclerosis includes a spectrum of intima-media thickening, plaque formation, stenosis, and eventually occlusion. Several stages of the atherogenic process have been described, starting from endothelial dysfunction, subendothelial accumulation of low-density lipoproteins (LDL), and chronic inflammation within the arterial wall leading to formation of plaque [2, 3].

Vitamin D is a group of fat soluble secosteroids which play an important role in musculoskeletal health by regulating calcium and phosphate metabolism [4]. There are two major forms of vitamin D; ergocalciferol (D_2_) and cholecalciferol (D_3_). The former is found in plants while the latter is found in humans and is synthesized following irradiation by ultraviolet B [5]. Both are converted to 25-hydroxyvitamin D (25(OH)D) by 25-hydroxylase (CYP2R1) in the liver and are activated by 1α-hydroxylase (CYP27B1) in the kidney to form 1,25-hydroxyvitamin D (1,25(OH_2_)D) [6]. Serum 25(OH)D assay is used to measure vitamin D status due to its higher serum concentration and longer half-life. In addition, a developing vitamin D deficiency is compensated for by increased activation of 1α-hydroxylase which results from increased secretion of PTH, thus restoring 1,25(OH_2_)D levels and thereby masking vitamin D deficiency [7].

The chronic kidney disease (CKD) population has a high prevalence of carotid atherosclerosis [8, 9] and vitamin D deficiency [10, 11]. Numerous observational studies on the association between serum 25(OH)D and carotid atherosclerosis have showed inconsistent results [2]. As for the CKD population, there have only been two studies; one demonstrated an inverse relationship [12] while the other showed mixed results [13]. Thus, we have aimed to examine the relationship between serum 25(OH)D levels and carotid atherosclerosis in CKD patients as measured by CCA-IMT and the presence of carotid plaque or stenosis.

## Methods

### Study design and sampling method

**Fig. 1:**
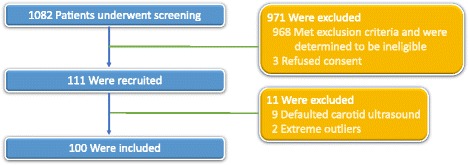
Flow diagram of enrolment

This was a cross sectional study conducted between September 2015 and March 2016. It has adhered to the STROBE guidelines for observational studies. A consecutive sampling methodology was used for participant recruitment. The flow diagram is shown in Fig. 1.

### Population

A total of 100 pre-dialysis CKD stage 3–4 patients who attended nephrology, diabetic, and outpatient clinics at the University of Malaya Medical Centre (UMMC) were included. Patients who were 70 years old and above, had underlying coronary artery disease, heart failure, malignancy, active infection, or were on immunosuppressive drugs or vitamin D supplements were excluded.

### Ethics approval and informed consent

Ethics approval (MECID 201412-878) was obtained from the UMMC Medical Ethics Committee which governs all research involving humans. Participants provided written informed consent prior to being enrolled in the study.

### Data collection procedures

Socio-demographic data, medical history, alcohol and smoking history, and medication history were obtained from all subjects. Body mass index (BMI) was calculated as weight in kilograms divided by height in meters squared (kg/m^2^). During the enrolment phase, previous biochemical tests were reviewed through the laboratory information system (TD-Web version 11.61B, Technidata SAS, France) and results within 2 weeks were exempted from repeat analysis. The biochemical analysis of blood samples was conducted in the same laboratory. The Chronic Kidney Disease Epidemiology Collaboration (CKD-EPI) formula was used to calculate the estimated glomerular filtration rate (eGFR). Participants were categorized into three CKD stages: stage 3A (45–59 mL/min per 1.73 m^2^), stage 3B (30–44 mL/min per 1.73 m^2^) and stage 4 (15–29 mL/min per 1.73 m^2^) according to Kidney Disease Outcome Quality Initiative (KDOQI) criteria.

### Biochemistry assay

Serum 25(OH)D and intact parathyroid hormone (PTH) levels were determined using direct chemiluminesent immunoassay (ADVIA Centaur XP; Siemens Healthcare Diagnostics, Tarrytown, New York, USA). The detection limit of the device was <10 nmol/L, with the intra- and inter-assay coefficient of variations of 4.2 and 11.9 % respectively. This assay demonstrated comparable performance with the reference standard, liquid chromatography tandem mass spectrometry (LC-MS/MS) [14–16]. A mean of 5 nmol/L was assumed for results reported as <10 nmol/L. Vitamin D status was defined as deficiency (< 25 nmol/L) [17] and non-deficiency (≥ 25 nmol/L). Serum calcium, inorganic phosphate, renal profiles and lipid profiles were measured using colorimetric methods (ADVIA 2400 Chemistry System; Siemens Healthcare Diagnostics, Tarrytown, New York, USA). Complete blood counts were analysed using fluorescent flow cytometry (XN-10; Sysmex, Kobe, Japan).

### Carotid ultrasound

An ultrasound system equipped with high-resolution, linear-array transducers in the frequency of 4–9 MHz (Philips iU22; Philips, Seattle, Washington, USA) was used. Carotid arteries were imaged with L9-3 and L8-4 transducers. It was performed by a single certified sonographer and images were verified by an experienced stroke neurologist. All participants underwent a carotid ultrasound within 2 weeks from 25(OH)D assay to measure intima-media thickness (IMT) and to assess the presence of carotid plaque or stenosis at the common carotid arteries (CCA), internal carotid arteries (ICA), and external carotid arteries (ECA). Three IMT measurements were obtained at the far walls of the distal, 2 cm from the common carotid artery. The mean of these three measurements was recorded as the IMT for that side (left and right). CCA-IMT was defined as the average of left and right IMT. The images were obtained at a 30° angle and the length of the segments measured was 1 cm. The image analysis was done using QLAB Vascular Ultrasound Quantification software, version 7.1. The analysis was done offline. The presence of carotid plaque or significant stenosis (≥ 50 %) in the CCA, bifurcation, and ICA bilaterally were documented. Abnormal CCA-IMT was defined as CCA-IMT ≥ 0.8 mm which is beyond the 75th percentile as per the American Society of Echography Task Force recommendations [18, 19]. Plaque was defined as a focal structure that encroaches into the arterial lumen of ≥ 0.5 mm or 50 % of the surrounding IMT value [20]. Significant stenosis was defined as peak-systolic velocities ≥ 125 cm/s and end-diastolic velocities ≥ 40 cm/s as recommended by the Society of Radiologists in Ultrasound [21].

### Statistical analysis

A statistical analysis was performed using the Statistical Package for Social Sciences for Windows, version 23.0 (IBM Corp., Armonk, New York, USA). Clinical characteristics data was expressed as means ± standard deviation (SD). Differences between groups were analysed using independent *t*-test and one-way analysis of variance (ANOVA) when the dependent variable was normally distributed, otherwise by Mann-Whitney U and Kruskal-Wallis H tests. Appropriate transformations were made when necessary to ensure normality or linearity assumptions were met. The correlation between serum 25(OH)D levels and CCA-IMT was determined using Pearson’s product-moment correlation coefficients. Linear regression analysis was performed to determine the predictors of serum 25(OH)D levels. Multiple linear regression models were used to identify the predictors of CCA-IMT, abnormal CCA-IMT, and plaque presence. Age, gender, race, BMI, smoking habits, diabetes status, hypertension status, eGFR, serum triglyceride, LDL cholesterol, HDL cholesterol, calcium, phosphate, PTH, and 25(OH)D levels were taken as independent variables for modelling. Backward elimination was applied to remove insignificant variables. All *P*-values were calculated two-sided and a value of < 0.05 was considered as significant.

## Results

### Demographic and clinical characteristics

A total of 100 CKD stage 3–4 patients were evaluated. The proportion of CKD patients at stage 3A was 29 %, stage 3B was 40 %, and stage 4 was 31 %. The mean age of the study population was 59 years. Fifty-seven percent of them were female. Ethnicity was comprised of 50 % Malay, 30 % Chinese, and 20 % Indian. The mean BMI was 28.16 kg/m^2^. The majority of the patients had hypertension (93 %), diabetes mellitus (76 %), and dyslipidaemia (79 %). The mean value of serum 25(OH)D levels was 39.83 ± 22.43 nmol/L. The participant’s characteristics were summarized in Table 1.

**Table 1:** Characteristics of participants

Variables	Participants (*n* = 100)
Age, years	59.05 ± 8.48
Male	43 (43.0)
Race, Malay/Chinese/Indian	50/30/20
Weight, kg	73.96 ± 16.94
Height, cm	162.03 ± 8.08
BMI, kg/m^2^	28.16 ± 5.77
Smokers	19 (19.0)
Diabetes mellitus	76 (76.0)
Hypertension	93 (93.0)
Dyslipidemia	79 (79.0)
Total cholesterol, mmol/L	4.44 ± 1.37
Triglyceride, mmol/L	1.81 ± 0.80
HDL cholesterol, mmol/L	1.20 ± 0.34
LDL cholesterol, mmol/L	2.44 ± 1.18
CKD, Stage 3A/3B/4	29/40/31
Urea, mmol/L	9.82 ± 3.43
Creatinine, mmol/L	167.62 ± 57.53
eGFR, mls/min/1.73 m^2^	36.49 ± 12.12
Hemoglobin, g/dL	11.96 ± 1.77
Ca^2+^, mmol/L	2.26 ± 0.11
PO_4_^2-^, mmol/L	1.25 ± 0.23
PTH, pmol/L	8.01 ± 7.09
25(OH)D, nmol/L	39.83 ± 22.43

Data in parentheses denote percentages

*25(OH)D* 25-hydroxyvitamin D, *BMI* body mass index, *CKD* chronic kidney disease, *eGFR* estimated glomerular filtration rate, *HDL* high-density lipoprotein, *LDL* low-density lipoprotein, *PTH* intact parathyroid hormone

A one-way ANOVA was conducted to determine the difference in mean serum 25(OH)D levels between various CKD stages. The mean levels for stage 3A, stage 3B, and stage 4 were 42.69 ± 20.68 nmol/L, 40.35 ± 24.42 nmol/L and 36.48 ± 21.60 nmol/L respectively. A Tukey post hoc analysis revealed that the differences between the groups were not significant (*P* = 0.558), as shown in Table 2.

**Table 2:** Mean serum 25(OH)D (nmol/L) according to stages of CKD

Stages of CKD	n	Mean (SE)	Mean difference (SE)	*P*-value (*t*-test)
3A	29	42.69 (3.83)	Ref	Ref
3B	40	40.35 (3.86)	2.34 (5.49)	0.905
4	31	36.48 (3.88)	6.21 (5.82)	0.537
*P*-value for Wald *F*			0.558	

*25(OH)D* 25-hydroxyvitamin D, *CKD* chronic kidney disease, *SE* standard error of mean, *Ref* reference group

### Demographic and clinical characteristics according to vitamin D status

Table 3 displays the detailed characteristics of participants according to their vitamin D status. Twenty-nine percent of the patients had vitamin D deficiency. Gender (*P* < 0.001), race (*P* = 0.002), smoking habits (*P* = 0.019), haemoglobin (*P* = 0.014), serum phosphate (*P* = 0.038), and serum PTH (*P* = 0.014) levels differed significantly by vitamin D status. The mean serum 25(OH)D levels for the vitamin D deficiency group was 13.76 ± 6.31 nmol/L while the non-deficiency groups was 50.48 ± 17.30 nmol/L.

**Table 3:** Characteristics of participants according to vitamin D status

Variable	Deficiency (*n* = 29)	Non-deficiency (*n* = 71)	*P*-value
Age, years	57.52 ± 8.10	59.68 ± 8.61	0.098
Gender			<0.001
Male	4 (13.8)	39 (54.9)	
Female	25 (86.2)	32 (45.1)	
Race			0.001
Malay	17 (58.6)	33 (46.5)	
Chinese	2 (6.9)	28 (39.4)	
Indian	10 (34.5)	10 (14.1)	
Weight, kg	75.56 ± 20.24	73.30 ± 15.51	0.764
Height, cm	161.24 ± 7.88	162.36 ± 8.19	0.534
BMI, kg/m^2^	28.92 ± 6.86	27.84 ± 5.28	0.401
Smokers	1 (3.4)	18 (25.4)	0.011
Diabetes mellitus	24 (82.8)	52 (73.2)	0.440
Hypertension	26 (89.7)	67 (94.4)	0.410
Dyslipidemia	23 (79.3)	56 (78.9)	1.000
Total cholesterol, mmol/L	4.81 ± 1.75	4.29 ± 1.16	0.111
Triglyceride, mmol/L	2.06 ± 0.97	1.70 ± 0.69	0.093
HDL Cholesterol, mmol/L	1.22 ± 0.40	1.19 ± 0.32	0.920
LDL Cholesterol, mmol/L	2.70 ± 1.53	2.33 ± 0.99	0.244
CKD			0.450
Stage 3A	6 (20.7)	23 (32.4)	
Stage 3B	12 (41.4)	28 (39.4)	
Stage 4	11 (37.9)	20 (28.2)	
Urea, mmol/L	9.18 ± 3.34	10.08 ± 3.46	0.196
Creatinine, mmol/L	165.21 ± 61.38	168.61 ± 56.30	0.587
eGFR, mls/min/1.73 m^2^	34.17 ± 11.58	37.44 ± 12.28	0.223
Hemoglobin, g/dL	11.28 ± 1.60	12.23 ± 1.77	0.014
Ca^2+^, mmol/L	2.26 ± 0.10	2.27 ± 0.11	0.678
PO_4_^2-^, mmol/L	1.31 ± 0.21	1.22 ± 0.23	0.038
PTH, pmol/L	11.49 ± 10.33	6.33 ± 3.97	0.014
25(OH)D, nmol/L	13.76 ± 6.31	50.48 ± 17.30	<0.001

Data in parentheses denote percentages

*25(OH)D* 25-hydroxyvitamin D, *BMI* body mass index, *CKD* chronic kidney disease, *eGFR* estimated glomerular filtration rate, *HDL* high-density lipoprotein, *LDL* low-density lipoprotein, *PTH* intact parathyroid hormone

### Predictors of Serum 25(OH)D Levels

Univariate linear regression analysis showed that age (*P* = 0.023), male gender (*P* < 0.001), Chinese ethnicity (*P* < 0.001), smoking habits (*P* = 0.001), diabetes mellitus (*P* = 0.004), haemoglobin (*P* < 0.001), serum triglyceride (*P* = 0.041), serum LDL Cholesterol (*P* = 0.018), and serum PTH (*P* = 0.045) levels were predictors of serum 25(OH)D levels. Chinese ethnicity was the strongest predictor among all, as shown in Table 4.

**Table 4:** Univariate linear regression for predictor of serum 25(OH)D levels

Variable	β	*P*-value
Age^a^	-0.228	0.023
Male	0.414	<0.001
Race		
Malay	Ref	Ref
Chinese	0.461	<0.001
Indian	-0.126	0.173
BMI	-0.068	0.501
Smokers	0.331	0.001
Diabetes mellitus	-0.283	0.004
Hypertension	0.116	0.252
Triglyceride^b^	-0.205	0.041
LDL Cholesterol^b^	-0.239	0.018
HDL Cholesterol^b^	-0.080	0.427
eGFR	0.108	0.283
Hemoglobin	0.397	<0.001
Ca^2+^	-0.023	0.826
PO_4_^2-^	-0.168	0.105
PTH^b^	-0.220	0.045

Linearity, normality of residuals, independence of residuals and homoscedasticity assumptions met. No significant outliers, high leverage points or highly influential points

*β* standardized coefficient, *Ref* reference group, *BMI* body mass index, *eGFR* estimated glomerular filtration rate, *HDL* high density lipoprotein, *LDL* low density lipoprotein, *PTH* intact parathyroid hormone

^a^ reflection of square root data transformation

^b^ logarithmic data transformation

### Carotid outcomes of participants

Among the participants, 23 % had abnormal CCA-IMT and 67 % had carotid plaque. Carotid stenosis was only found in three subjects within the entire cohort, as shown in Table 5. Carotid outcomes were not significantly different among CKD stages.

**Table 5:** Carotid outcomes of participants

Variable	Participants (*n* = 100)
Abnormal CCA-IMT	23 (23.0)
CCA-IMT, mm	0.724 ± 0.227
Left CCA, mm	0.731 ± 0.244
Right CCA, mm	0.711 ± 0.265
Plaque	67 (67.0)
Left CCA	47 (47.0)
Bulb	47 (47.0)
Proximal	2 (2.0)
Middle	1 (1.0)
Distal	5 (5.0)
Left ICA	6 (6.0)
Left ECA	1 (1.0)
Right CCA	50 (50.0)
Bulb	49 (49.0)
Proximal	1 (1.0)
Middle	0 (0.0)
Distal	4 (4.0)
Right ICA	10 (10.0)
Right ECA	3 (3.0)
Stenosis	3 (3.0)

Data in parentheses denote percentages

*CCA-IMT* common carotid artery intima-media thickness, *CCA* common carotid artery, *ECA* external carotid artery, *ICA* internal carotid artery

### Carotid outcomes of participants according to vitamin D status

The vitamin D deficiency and non-deficiency groups did not differ significantly in terms of abnormal CCA-IMT (*P* = 0.443), carotid plaque (*P* = 0.349), and carotid stenosis (*P* = 0.554), as shown in Table 6.

**Table 6:** Carotid outcome according to vitamin D status

Variable	Deficiency (*n* = 29)	Non-deficiency (*n* = 71)	*P*-value
Abnormal CCA-IMT	5 (17.2)	18 (25.4)	0.443
CCA-IMT, mm	0.679 ± 0.144	0.742 ± 0.252	0.356
Left CCA, mm	0.685 ± 0.195	0.750 ± 0.261	0.282
Right CCA, mm	0.668 ± 0.165	0.729 ± 0.295	0.566
Plaque	17 (58.6)	50 (70.4)	0.349
Left CCA	11 (37.9)	36 (50.7)	0.276
Bulb	11 (37.9)	36 (50.7)	0.276
Proximal	0 (0.0)	2 (2.8)	1.000
Middle	0 (0.0)	1 (1.4)	1.000
Distal	0 (0.0)	5 (7.0)	0.318
Left ICA	3 (10.3)	3 (4.2)	0.352
Left ECA	0 (0.0)	1 (1.4)	1.000
Right CCA	14 (48.3)	36 (50.7)	1.000
Bulb	13 (44.8)	36 (50.7)	0.662
Proximal	1 (3.4)	0 (0.0)	0.290
Middle	0 (0.0)	0 (0.0)	-
Distal	1 (3.4)	3 (4.2)	1.000
Right ICA	0 (0.0)	10 (14.1)	0.059
Right ECA	1 (3.4)	2 (2.8)	1.000
Stenosis	0 (0.0)	3 (3.0)	0.554

Data in parentheses denote percentages

*CCA-IMT* common carotid artery intima-media thickness, *CCA* common carotid artery, *ECA* external carotid artery, *ICA* internal carotid artery

### Association between Serum 25(OH)D Levels and Carotid Atherosclerosis

#### Correlation between Serum 25(OH)D Levels and CCA-IMT

**Fig. 2:**
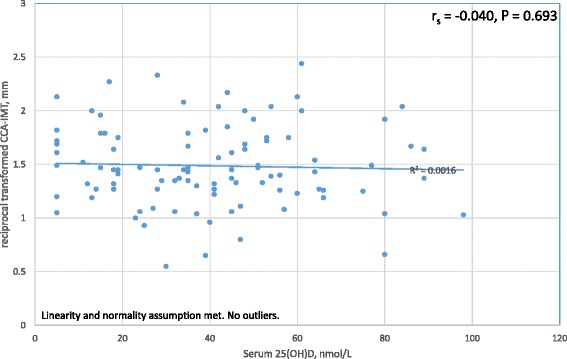
Correlation between serum 25(OH)D levels and CCA-IMT

Pearson’s product-moment correlation was used to examine the relationship between serum 25(OH)D levels and CCA-IMT. Figure 2 shows that no significant correlation (*P* = 0.693) was found.

#### Predictors of CCA-IMT, abnormal CCA-IMT, and plaque presence

Multiple linear regression analyses were performed to assess the association between serum 25(OH)D levels and CCA‐IMT, abnormal CCA‐IMT, and plaque presence. A backward elimination method adjusting for potential confounders namely age, gender, race, BMI, smoking habits, diabetes status, hypertension status, eGFR, serum triglyceride, LDL cholesterol, HDL cholesterol, calcium, phosphate, and PTH levels was applied. No significant association between serum 25(OH)D levels and CCA-IMT, abnormal CCA-IMT, and plaque presence was found. Notably, smoking habits was significantly associated with CCA-IMT (*P* = 0.003) and it predicted abnormal CCA-IMT (*P* = 0.003). Chinese ethnicity predicted abnormal CCA-IMT (*P* = 0.012) and plaque presence (*P* = 0.020) whilst serum PTH levels predicted plaque presence only. Table 7 displays the final step of each regression model.

**Table 7:** Backward multiple linear regression for predictor of CCA-IMT^a^, abnormal CCA-IMT and plaque presence

Variable	B	SE for B	β	95 % CI for B	*P*-value
CCA-IMT^a^					
Chinese	0.097	0.055	0.192	-0.012–0.206	0.079
Smoker	0.201	0.064	0.329	0.073–0.330	0.003
Diabetes	0.112	0.057	0.213	-0.001–0.225	0.051
Abnormal CCA-IMT					
Chinese	0.289	0.113	0.317	0.064–0.514	0.012
Smoker	0.369	0.121	0.335	0.127–0.610	0.003
25(OH)D	-0.004	0.002	-0.235	-0.009-<0.001	0.073
Plaque Presence					
Chinese	0.265	0.112	0.257	0.043–0.488	0.020
PTH^a^	0.325	0.158	0.222	0.010–0.641	0.043

Age, gender, race, BMI, smoker, diabetes status, hypertension status, eGFR, serum triglyceride, LDL cholesterol, HDL cholesterol, calcium, phosphate, PTH, and 25(OH)D levels were included in the model. Linearity, normality of residuals, independence of residuals and homoscedasticity assumptions met. No multicollinearity problem. No significant outliers

*B* unstandardized regression coefficient, *SE* standard error of the coefficient, *β* standardized coefficient *CI* confidence interval, *25(OH)D* 25-hydroxyvitamin D, *PTH* intact parathyroid hormone

^a^ logarithmic data transformation

## Discussion

The inverse relationship between serum 25(OH)D levels and cardiovascular diseases has been observed in epidemiological studies. However, no conclusions can be draw on the causality of this relationship. In fact, interventional studies have failed to show that vitamin D-raising interventions decreased cardiovascular events [22, 23]. The main findings of our study are: (1) no difference in CCA-IMT between vitamin D deficient and non-deficient patients; (2) no correlation between serum 25(OH)D levels and CCA-IMT; and (3) no relationship between serum 25(OH)D levels and CCA-IMT, abnormal CCA-IMT, and plaque presence in multivariate analysis which corroborates the findings from interventional studies.

Contrary to our findings, Yadav et al. reported that serum 25(OH)D levels were inversely associated with CCA-IMT in CKD patients [12]. Notably, their study participants had more severe (stage 4 and 5) CKD stages. Our study has similar percentage of carotid plaque with a study by Gracia et al. [13]. Again, no association between serum 25(OH)D levels and carotid plaque were observed. Instead, serum PTH levels predicted carotid plaque presence. To the best of our knowledge, PTH has only been reported to be associated with vascular calcification in animal model [24] and arterial stiffness in both diabetic patients [25] and postmenopausal women [26].

The link between vitamin D concentrations and carotid atherosclerosis was not clear, even in non-CKD population. Blondon et al. (*n* = 3251) reported that both vitamin D and PTH had no significant impact on carotid IMT and plaque in a multi-ethnic cohort with high cardiovascular risk [27]. Similarly, Deleskog et al. (*n* = 3430) found no consistent association between serum 25(OH)D and carotid IMT in a high risk European population [28].

There are several explanations for the disparate results observed. Firstly, a longitudinal study has showed that CKD progression was independently associated with atheromatosis progression [13]. Serum 25(OH)D levels also fall with eGFR decline and may therefore result in false association with atherosclerosis. Secondly, the demographic profiles of enrolled subjects were different. Different latitudes receive different amounts of sunlight, resulting in a different range of baseline serum 25(OH)D levels and carotid IMT. Countries that are far from the equator might have a seasonal variation of serum 25(OH)D concentrations and the impact of nadir serum 25(OH)D on carotid IMT might remain despite recovery of serum 25(OH)D levels. Thirdly, the relationship between vitamin D and carotid IMT was perhaps U-shaped, rather than linear [29, 30]. Recently, Van Dijk et al. suggested that the dip of a U-shaped relationship is at serum 25(OH)D levels of 50 nmol/L [29]. This might support the findings of Melamed et al. who proposed a similar U-shaped relationship between serum 25(OH)D and all-cause mortality in participants of the Third National Health and Examination Survey (NHANES III) [31]. In Blondon’s study [27], two thirds of the participants had baseline serum 25(OH)D ≥ 50 nmol/L, while most participants in Deleskog’s study [28] had serum 25(OH)D between 25 and 75 nmol/L. Analysis of these populations as a whole might attenuate the relationship between serum 25(OH)D and carotid atherosclerosis.

The observed non-linear relationship can be explained by the dualistic roles of vitamin D. It has both protective and harmful effects on vascular health. The anti-inflammatory and immune-modulatory properties are protective against atherosclerosis. It decreases endothelial oxidative stress, improves vascular muscle tone, inhibits formation of foam cells, regulates proliferation and migration of vascular smooth muscle cells (VSMC), inhibits release of pro-inflammatory cytokines, and suppresses pro-atherogenic T lymphocytes [3, 5, 32]. On the other hand, it may up-regulate vitamin D receptor (VDR) expression inducing osteogenic differentiation and mineralization of VSMC, promote production of matrix metalloproteinases leading to vascular remodelling, down-regulate calcification inhibitor fetuin-A expression, and increase calcium-phosphate product [33–36].

The renal expression of 1α-hydroxylase is regulated by serum calcium, phosphate, parathyroid hormone (PTH), and fibroblast growth factor 23 (FGF23) levels. FGF23 is a hormone secreted from osteocytes in coordination with α-Klotho to promote renal phosphate excretion [37]. FGF23 levels increase at early CKD stages which results in a decrease of serum α-Klotho and 1,25(OH_2_)D levels [38]. Low levels of circulating 1,25(OH_2_)D via a negative feedback mechanism causes a rise of PTH concentrations to stimulate reabsorption of calcium, bone resorption and 1,25(OH_2_)D production. PTH also increases total collagen synthesis and reorganizes collagen in VSMC leading to vascular stiffness [39, 40]. In addition to secondary hyperparathyroidism, FGF23 excess and Klotho deficiency have been recognized as novel factors contributing to vascular calcification [41–43] which might in part explain the link between CKD progression and atherosclerosis. In a recent breakthrough, Chang et al. demonstrated the ability of intermedin_1-53_ to increase α-Klotho levels which can suppress vascular calcification and delay the progression of CKD [44]. Both vascular calcification and elastin fibres degeneration cause arterial stiffening [35] which may increase risk of vessel wall damage and result in atherosclerosis [45]. Hence, α-Klotho deficiency plays a pivotal role in accelerated atherosclerosis.

The strength of our study was the inclusion of a more comprehensive carotid plaque assessment rather than solely relying on a carotid IMT measurement. Our study had several limitations: (1) it included a relatively small number of patients thus may have a lack of statistical power; (2) it was a single-centre study so the results cannot be generalised; and (3) other confounding factors, such as α-Klotho and FGF23 were not taken into account.

## Conclusion

Our study showed that there was no important association between serum 25(OH)D and CCA-IMT.

## Abbreviations

1,25(OH2)D: 1,25-hydroxyvitamin D
25(OH)D: 25-hydroxyvitamin D
BMI: Body mass index
CCA: Common carotid artery
CCA-IMT: Common carotid artery intima-media thickness
CKD: Chronic kidney disease
ECA: External carotid artery
eGFR: Estimated glomerular filtration rate
FGF23: Fibroblast growth factor 23
HDL: High density lipoprotein
ICA: Internal carotid artery
IMT: Intima-media thickness
LDL: Low density lipoprotein
PTH: Parathyroid hormone
UMMC: University of Malaya Medical Centre
VDR: Vitamin D receptor
VMSC: Vascular smooth muscle cells
